# Prevalence and Incidence of HCV Infection among Prisoners in Central Brazil

**DOI:** 10.1371/journal.pone.0169195

**Published:** 2017-01-06

**Authors:** Marco Antonio Moreira Puga, Larissa Melo Bandeira, Mauricio Antonio Pompilio, Julio Croda, Grazielli Rocha de Rezende, Luiz Fernando Paiva Dorisbor, Tayana Serpa Ortiz Tanaka, Gabriela Alves Cesar, Sheila Araújo Teles, Simone Simionatto, Alisson Richard Teixeira Novais, Bruna Nepomuceno, Lisie Souza Castro, Barbara Vieira do Lago, Ana Rita Coimbra Motta-Castro

**Affiliations:** 1 Federal University of Mato Grosso do Sul, Campo Grande, MS, Brazil; 2 Oswaldo Cruz Foundation, Mato Grosso do Sul, MS, Brazil; 3 Federal University of Grande Dourados, Dourados, MS, Brazil; 4 School of Nursing, Federal University of Goiás, Goiânia, GO, Brazil; 5 Oswaldo Cruz Foundation, Rio de Janeiro, RJ, Brazil; Centers for Disease Control and Prevention, UNITED STATES

## Abstract

The aim of this multicenter, cross sectional study was to assess the prevalence, incidence and associated risk factors among incarcerated populations from twelve Brazilian prisons. The total of 3,368 individuals from twelve prisons was randomly recruited between March 2013 and March 2014. Participants were interviewed, and provided blood samples which were tested for antibodies to Hepatitis C (HCV ab). One year after the first investigation, a cohort study was conducted with 1,656 inmates who participated the cross sectional study. Positive samples were tested for the presence of HCV RNA. Out of 3,368 inmates, 520 (15.4%) were females, and 2,848 (84.6%) were males. The overall prevalence of HCV was 2.4% (95% CI: 1.9 to 2.9), with 0.6% (95% CI: 0.4 to 0.8) in females, and 2.7% (95% CI: 2.1 to 3.3) in males (p<0.01). HCV RNA was detected in 51/80 (63.7%) samples. Among men prisoners, multivariate analysis of associated factors showed independent associations between HCV exposure and increasing age, inject drug use, length of incarceration, smoking hashish, sharing needle and syringe and HIV positivity. During the cohort study, 7/1,656 new cases of HCV infection were detected, and the incidence rate was 0.4/100 person-year. Once high frequency rates of specific HCV risk behaviors and new HCV infections have been identified inside prisons, effective interventions strategies such as screening, clinical evaluation and treatment to reduce the spread of HCV infection are essential.

## Introduction

Hepatitis C virus (HCV) infection is one of the major public health problem worldwide, with approximately 170 million people infected [1,2]. In Brazil it is estimated that 1% to 2% of general population is infected with HCV [3].

Worldwide, more than 10 million people are incarcerated in jails and prisons. Brazil’s number at 607,731 prisoners, has tripled nationwide over the last 20 years and ranks fourth in the world in relation to the rate of prison population [4,5]. The prison population is considered at high risk of acquiring infectious diseases related to confined conditions due to behavioral factors related to injection drug use, needle-sharing, non-professional tattooing and unprotected sexual activity. These risky behaviors may precede imprisonment and frequently continue during incarceration [6,7].

The Mato Grosso do Sul state is uniquely positioned in the Central-West region of Brazil on the border of Bolivia and Paraguay allows advantageous geography for trafficking in illegal drugs. Also, this State is left with the proportionally largest prison population in the country, concentrating 13,000 inmates, which is double its capacity. There are more prisons built in this area probably due to the high rates of drug-trafficking crimes [8,9].

Given the lack of prevention policy on HCV infection in Brazilian prisons, the aim of this study was to estimate the prevalence, incidence rates, and predictive factors of HCV infection among prison inmates from 12 prisons in Midwest Brazil.

## Methods

The cross-sectional survey was carried out between March 2013 and March 2014. Based on the information from the State Agency of the Administration of Prisons, at the time of our study there were about 9,913 inmates at 21 closed penal institutions. In the “closed” subset of the system, the prison time is enforced in a penitentiary, and the inmate is at all time subjected to supervision.

**Fig 1:**
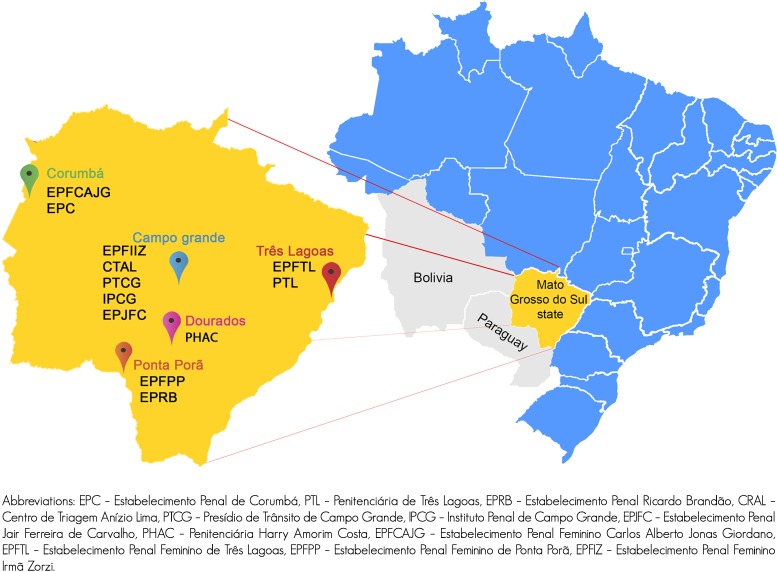
Geographical location of study prisons, Mato Grosso do Sul, Brazil (Adapted from reference 10).

**Fig 2:**
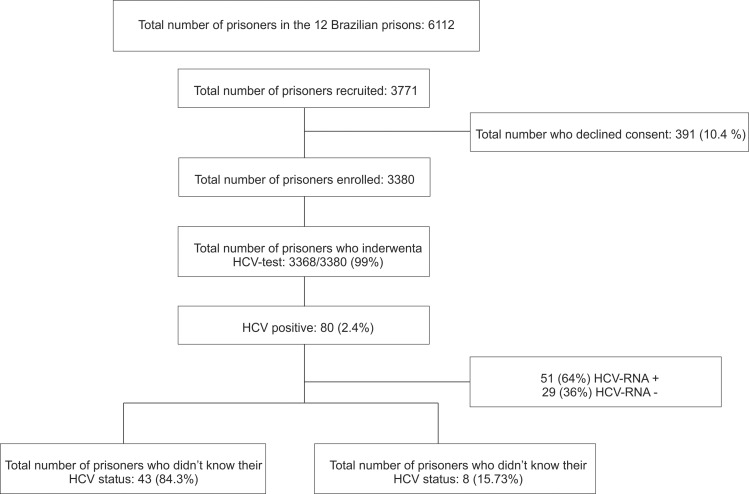
Flow chart of the recruitment of the study and screening process for HCV.

Twelve of the 21 closed high security prisons in the 5 largest cities in the state (Campo Grande, Corumbá, Dourados, Ponta Porã and Três Lagoas) were included (Fig 1) [10]. The sample size was calculated based on the expected 2% prevalence of HCV with a variation of 1%, power of 80% and alpha-type error of 5%. The study population is 7,221 prisoners, and the sample size was 3,159 prisoners. We added 20% more individuals (total, 3,771 prisoners) to account for anticipated loss due to refusal to participate (Fig 2). Proportional stratified sampling was performed using each prison as a unit of randomization. On the data collection day, the prisoners were ordered numerically in ascending order from the lists provided by the prison, and a list of random numbers was generated using the Epi-Info 6.04 software (Atlanta, GA, USA). In order to be eligible to participate, patients had to be (a) aged 18 years or older (b) in prison custody (c) able to consent for themselves (d) suitable to be interviewed by a researcher alone (no risk markers), and able to understand spoken Portuguese. This study was conducted with the approval of the research ethics committee at the Universidade Federal da Grande Dourados, under protocol number 191.877 CAAE 05598912.00000.5160. All eligible participants provided written informed consent prior to participation. Participation in the study was voluntary and no compensation was provided. The treatment offered to individuals who did not participate the study was the same given to participants.

One year after the first investigation (2014–2015), a cohort study was conducted with 1,656 inmates who participated the cross sectional study using the following individuals inclusion criteria: (a) not leave prisons (for any reason) during this period; (b) accepting to be interviewed regarding pre-defined risk-factors associated with reinfection; (c) being susceptible to HCV infection (anti-HCV negative).

An interview was conducted through a questionnaire on socioeconomic and demographic characteristics (personal data) at the moment of recruitment. The variables obtained during the interview included age, sex, marital status, educational level, drug use over the last year, sexual history, history of sexually transmitted infections (STIs), history of blood transfusion, tattoos, piercings, previous surgery, self-reported mental illness, previous incarceration and time in prison. The participant’s race/ethnicity (i.e., white, black, asian or multiracial) was self-reported. An anonymous identification number was used to cross-reference information contained in the questionnaires.

HCV exposure was considered present in individuals who were positive for anti-HCV by enzyme immunosorbent assay (ELISA–Murex Diagnostics, UK) and confirmed by “line imunoassay” (INNO-LIA III HCV Ab, Innogenetics, Belgic). Positive samples were submitted to detection of HCV RNA by Real Time HCV assay (qPCR) (Abbott RealTime HCV^®^). The anti-HIV-1/2 was determined using enzyme immunosorbent assay and confirmed by Western Blot assay as described by Sgarbi et al., 2015 [11].

The variables evaluated were double entered and checked by Research Electronic Data Capture (REDCap) online database. The processing and analysis were performed using the statistical program SPSS Statistical Software version 15 and STATA version 13. Prevalence rates were calculated with 95% confidence interval. Samples were analyzed with the chi-square test to assess the difference in risk factors between male and female inmates. Univariate analysis was used to assess the association between independent and dependent variables. Variables that were significant (p <0.10) were included in the multivariate analysis by robust Poisson regression model to verify the joint action of possible risk factors. After adjustment, independent variables that maintained association with HCV exposure (p ≤ 0.05) were kept in the model. Incidence rates were calculated using person-years which was followed during a one year follow-up study.

## Results

Of the 3,771 prisoners randomly selected to participate, 3,368 (89.3%) agreed to be interviewed and also provided biological samples. Of these, 301 (10.4%) individuals declined to participate the study, and 12 (0.3%) refused blood collection (Fig 2). Out of 3,368, 520 (15.4%) were female (mean age of 31.1 years) and 2,848 (84.6%) were male (mean age of 31.6 years). Sociodemographic characteristics, risk factors, prison variables and prevalence of HCV are presented in Table 1, according to gender. Significant differences between male and female prisoners were observed for almost all variables, highlighting the importance of analyzing these prisoners separately.

**Table 1:** Socio demographic characteristics, risk factors, prison variables and HCV infection among male and female prisoners at baseline (n = 3,368).

	Sex (Number/percentage)				
Variables	Malen = 2,848 (84.6%)	Missing n	Female (n = 520/15.4%)	Missing n	p—value
**HCV positive**	77 (2.7)	-	3 (0.6)	-	**<0.01**
**Sociodemographic**					
Age, years, mean±SD	32±10	-	32±10	-	0.36
Marital status, single	1,308(46)	21	173(34)	13	**<0.01**
**Ethnicity**		116		51	
White	916(33)		138(29)		**<0.01**
Multiracial	1,369(52)		283(62)		
Black	378(13)		35(7)		
Asian	69(2)		13(2)		
**Naturality in MS**	1,880(66)	-	276(53)	-	**<0.01**
**Schooling less than 4 years**	1,186(43)	66	281(55)	5	**<0.01**
**Drug History**					
**Non-IDU**					
Over the last year	1,536(54)	-	199(38)	-	**<0.01**
**IDU**					
Over the last year	26(1)	91	5(1)	16	0.91
Ever shared needles/syring	79(3)	14	28(5)	3	**<0.01**
**Sexual History**					
Sexual Preference		15		12	**<0.01**
- Heterosexual	2,786(98)		450(89)		
- Homosexual	47(2)		58(11)		
Previously had homosexual intercourse	153(5)	77	125(26)	45	**<0.01**
Ever had sex with an non-IDU	951(34)	13	256(50)	3	**<0.01**
Ever had sex with an IDU	82(3)	218	30(6)	25	**<0.01**
Stable Partner	1,433(50)	4	241(46)	-	0.10
Condom use		12		8	0.35
- Always	957(34)		162(32)		
- Sometimes/never	1,879(66)		350(68)		
**Other Risk Behavior**					
History of STI(s)	341(13)	157	51(10)	11	0.09
Episode of genital ulcer	63(2)	7	11(2)	1	0.89
Blood Transfusion	352(13)	49	59(11)	3	0.47
Tattoos	1,907(67)	9	324(62)	-	**0.03**
Piercings	189(7)	39	186(36)	2	**<0.01**
Shared Sharp Objects	1,020(36)	12	267(52)	1	**<0.01**
Surgery	1,115(39)	20	327(64)	4	**<0.01**
**HIV status**					
Positive	44 (1.54)	-	10 (1.92)	-	0.53
**Prison**					
Previous Incarceration	1,748(62)	25	207(40)	2	**<0.01**
Time in prison, months, mean±SD	20±27	-	12±12	-	**<0.01**
**Penal institutions**					**<0.01**
EPFCAJG	-		81(16)		
EPFTL	-		78(15)		
EPFPP	-		93(18)		
EPFIIZ	-		268(51)		
EPC	260(9)		-		
PTL	282(10)		-		
EPRB	250(9)		-		
CTAL	118(4)		-		
PTCG	286(10)		-		
IPCG	516(18)		-		
EPJFC	601(21)		-		
PHAC	535(19)		-		

MS- Mato Grosso do Sul state, IDU- Injection Drug Use, STI- Sexually Transmitted Infection, EPFCAJG- Estabelecimento Penal Feminino Carlos Alberto Jonas Giordano, EPFTL- Estabelecimento Penal Feminino de Três Lagoas, EPFPP- Estabelecimento Penal Feminino de Ponta Porã, EPFIIZ- Estabelecimento Penal Feminino Irmã Irma Zorzi, EPC- Estabelecimento Penal de Corumbá, PTL- Penitenciária de Três Lagoas, EPRB- Estabelecimento Penal Ricardo Brandão, CTAL- Centro de Triagem Anízio Lima, PTCG- Presídio de Trânsito de Campo Grande, IPCG- Instituto Penal de Campo Grande, EPJFC- Estabelecimento Penal Jair Ferreira de Carvalho, PHAC- Penitenciária Harry Amorim Costa.

Out of 3,368 participants, most were youths, had no more than primary education (65.8%), and were born in Mato Grosso do Sul state. Of these, 199 females (38%) and 1,536 (54%) male inmates were non-injectable drug users (p<0.01). Low frequency (1.0%) of inmates reported a history of injection drug use (IDU). Use of smoked marijuana was frequently reported (46.7%) among males, followed by the use of cocaine paste (intermediary product of the cocaine preparation process) (29.7%), hashish (17.3%), crack (6.9%), and heroin smoking (1.1%).

Sharing needles and syringes was reported only by 3.2% of the participants and were more frequent among female than male prisoners (p<0.01). Both prisoners reported high frequency (68 vs. 66%) of irregular condom use with no difference between the rates. Same sex intercourse was more common among females than males (26% vs. 5%; p<0.01). The mean period of current imprisonment and previously incarceration differed substantially from group to group (p<0.01).

The prevalence of HCV exposure found was 2.4% (80/3,368; 95% CI 1.91–2.95), ranging from 0.7% in PTCG (Presídio de Trânsito de Campo Grande) prison to 4.6% in IPCG (Instituto Penal de Campo Grande) (p<0.001), both located in Campo Grande city. HCV prevalence among females (0.6%; 95% CI 0.20–1.68) was lower than among males (2.7%; 95% CI 2.17–3.37; p = 0.003). The small sample size of anti-HCV positive cases among female inmates limited our ability to detect statistically significant risk factors.

HCV infection was significantly associated among male inmates with age over 30 years, length of incarceration, alcohol consumption, smoking crack, heroin and hashish, injection drug use, needle and syringe sharing, history of blood transfusion, STI, surgery, HIV positivity and more than five sexual partners in the last five years (p<0.05). These variables were included in a multivariate robust Poisson regression model. Age over 40 years, injection drug use, length of incarceration, needles and syringe sharing, HIV positivity and smoking hashish were independently associated with HCV exposure (Tables 2 and 3).

**Table 2:** Univariable regression analysis of risk factors associated with HCV prevalence among male prisoners at baseline (n = 2,848).

	Male				
	HCV (n = 77)				
Variables	Pos/Total	Missing	%	PR* (95% CI)**	p- value
**Age (years)**		1			
≤30	11/1,546	-	0.7	1.00	
31–40	13/770	-	1.7	2.37 (1.06–5.29)	**0.035**
41–50	32/335	-	9.6	13.42 (6.77–26.63)	**<0.001**
>50	20/147	-	13.6	19.12 (9.16–39.91)	**<0.001**
**Education (years)**		2			
≤ 9	60/2,027	-	4.7	1.00	
10–12	14/653	-	4.3	0.76 (0.33–1.77)	0.532
>12	1/66	-	2.0	0.65 (0.09–4.73)	0.675
**City of prison last**		-			
Dourados	10/535	-	1.9	1.00	
Campo Grande	47/1,520	-	3.1	1.65 (0.84–3.27)	0.148
Três Lagoas	6/283	-	2.1	1.13 (0.41–3.12)	0.807
Corumbá	6/260	-	2.3	1.23 (0.45–3.40)	0.683
Ponta Porã	8/250	-	3.2	1.71 (0.67–4.34)	0.257
**Lenght of Incarceration (months)**		3			
≤24	24/1,393	-	1.7	1.00	
25–48	7/617	-	1.1	0.66 (0.28–1.53)	0.331
49–72	13/312	-	4.2	2.42 (1.23–4.75)	**0.010**
>72	30/396	-	7.6	4.40 (2.57–7.52)	**<0.001**
**Alcohol consumption (last year)**		1			
No	46/1,295	-	3.6	1.00	
Yes	30/1,500	-	2.0	0.56 (0.35–0.89)	**0.014**
**Marijuana use in last year**		2			
No	37/1,491	-	2.5	1.00	
Yes	38/1,308	-	2.9	1.17 (0.74–1.84)	0.495
**Cocaine use in last year**		3			
No	49/1,959	-	2.5	1.00	
Yes	25/827	-	3.0	1.21 (0.75–1.96)	0.441
**Crack use in last year**		4			
No	63/2,569	-	2.5	1.00	
Yes	10/193	-	5.2	2.11 (1.08–4.12)	**0.028**
**Heroin use (smoke) in last year**		4			
No	69/2,726	-	2.5	1.00	
Yes	4/31	-	12.9	5.09 (1.86–13.96)	**0.002**
**Hashish use in last year**		5			
No	51/2,279	-	2.2	1.00	
Yes	21/476	-	4.4	1.97 (1.19–3.28)	**0.009**
**Injection drug use in last year**		4			
No	66/2,729	-	2.4	1.00	
Yes	7/28	-	25.0	10.33 (4.74–22.52)	**<0.001**
**Ever shared needles/syring**		-			
No	68/2,754	-	2.5	1.00	
Yes	9/80	-	11.3	4.55 (2.27–9.13)	**<0.001**
**Shared sharp objects**		1			
No	47/1,814	-	2.6	1.00	
Yes	29/1,022	-	2.8	1.09 (0.69–1.74)	0.700
**Piercings**		2			
No	73/2,620	-	2.8	1.00	
Yes	2/189	-	1.1	0.38 (0.09–1.55)	0.177
**Tattooing**		-			
No	29/920	-	3.2	1.00	
Yes	48/1,923	-	2.5	0.79 (0.50–1.25)	0.321
**Blood transfusion**		-			
No	61/2,441	-	2.5	1.00	
Yes	16/358	-	4.5	3.75 (1.51–9.30)	**0.004**
**Surgery history**		-			
No	32/1,704	-	1.9	1.00	
Yes	45/1,124	-	4.0	2.13 (1.35–3.35)	**0.001**
**History of STI(s)**		4			
No	52/2,345	-	2.2	1.00	
Yes	21/345	-	6.1	2.74 (1.65–4.56)	**<0.001**
**Ever had sex with an IDU**		4			
No	68/2,547	-	2.7	1.00	
Yes	5/83	-	6.0	2.26 (0.91–5.60)	0.08
**Previously had homosexual intercourse**		3			
No	70/2,618	-	2.7	1.00	
Yes	4/153	-	2.6	0.98 (0.36–2.68)	0.965
**Number of sexual partners in the last five years**		7			
≤1	33/874	-	3.8	1.00	
2–5	24/899	-	2.7	0.71 (0.42–1.20)	0.191
>5	13/907	-	1.4	0.38 (0.20–0.72)	**<0.003**
**Condom Use**		1			
Always	24/958	-	2.5	1.00	
Sometimes	27/1,088	-	2.5	0.99 (0.57–1.71)	0.973
Never	25/792	-	3.2	1.26 (0.72–2.20)	0.419
**HIV positive**	5/44		11.1	4.74 (1.82–12.36)	**<0.01**

*PR: prevalence ratio

**CI: confidence interval

STI: sexually transmitted infections; IDU: injecting drug users; if p≤ 0.05, there is significant statistical difference between values.

**Table 3:** Multivariable regression analysis of risk factors associated with HCV infection in male prisoners at baseline (n = 77).

	HCV (n = 77)	
Variables	PR* (95% CI)**	p- value
**Age (years)**		
≤30	1.00	
31–40	2.08 (0.78–5.57)	0.144
41–50	11.03 (4.55–26.71)	**<0.001**
>50	17.04 (6.77–42.85)	**<0.001**
**Length of Incarceration (months)**		
≤48	1.00	
49–72	2.53 (1.29–4.93)	**0.007**
>72	2.40 (1.36–4.26)	**0.003**
**History smoking hashish**	2.14 (1.07–4.27)	**0.030**
**IDU over the last year**	5.13 (1.97–13.37)	**0.001**
**Ever shared needles/syring**	2.58 (1.14–5.83)	**0.023**
**HIV positive**	3.33 (1.02–10.88)	**0.046**

*PR: prevalence 0072atio

**CI: confidence interval

IDU: Injection drug use. Adjusted by age, length of incarceration, number of prisoners per cell, alcohol consumption, smoking crack, heroin and hashish, injection drug use, needle and syringe sharing, history of transfusion, STD and surgery, number of sexual partners in the last five years.

The presence of HCV RNA was detected in 51 out of 80 anti-HCV positive samples (63.7%) by amplification of the NS5B region through RT-nested PCR.

During cohort study, 1,656 prisoners susceptible to HCV infection were submitted to interview and new blood samples were collected. Among them, 7 new cases of HCV infection were detected and the incidence rate was 0.4/100 person-year. The presence of HCV RNA was detected in 1 out of 7 anti-HCV positive samples. Most HCV incidence cases were among males (5/7) incarcerated in Campo Grande prisons and the mean age was 36 years. Risks behaviors found in 3 out of 7 new cases included inhaled cocaine use, tattooing inside the prison and history of sharing cutting instruments.

## Discussion

This study has important implications for public health. The prevalence of HCV exposure of 2.4% (95% CI 1.9–2.9) was approximately two times higher than among the general population (1.38%) and 14 times higher than among the blood donors (0.17%) of Campo Grande, Mato Grosso do Sul [12,13].

Otherwise, this prevalence is not consistent with previous studies conducted among inmates in Brazil, such as in Sergipe (3.1%), São Paulo (9.0%) and Campo Grande, MS, (4.8%) and higher than in Espírito Santo (1.0%) [14–17]. Although this prevalence is greater than the prevalence in the general population, the low prevalence found may be due to current low prevalence of HCV exposure in the Midwest region. Additionally, the difference between the prevalence of HCV exposure found in this study compared with the study conducted in the same region by Pompilio et al. (2011) may be explained by the decline in the frequency of injection drug users (10.6% to 1%) [16]. In fact, in recent years, the pattern of drug use in Brazil has changed, resulting in a smaller proportion of IDU which was largely replaced by smoked or inhaled crack cocaine [18–20].

When compared to international studies, the prevalence of HCV found in our study was lower than in California (34.3%), Indonesia (34.1%), Italy (22.4%), Iran (7.4%) and Lebanon (3.4%), which could be explained by the low frequency of injecting drug users (1%) in male and female prisoners [21–24]. In addition to that, there is a law prohibiting the use of illicit drugs in Brazil, inside and outside prisons, leading those who use drugs illegally in prisons to punishment such as increased imprisonment time [25].

There was a significant difference in HCV exposure between male and female prisoners (p<0.01), with a higher prevalence among males (2.7%) than females (0.6%). This result is similar to those observed by Flisiak et al. (2011) in Poland, probably because males are more exposed to risk factors for infection with hepatitis C than females [26]. Despite comparable rates of reported IDU, a higher percentage of male prisoners reported previous incarceration (62% vs. 40%; p<0.01) and mean (±SD) of length of incarceration (20±27 vs. 12±12 months; p<0.01). This result differs from other studies that reported a predominance of HCV exposure among female inmates [27–29].

Increasing age was associated with HCV among male inmates, probably due to prolonged exposure to HCV infection presenting risk behaviors throughout life, which was observed in other vulnerable population groups such as health care professionals, homeless people, drug users, as well as inmates [16,30–34].

In this study, injection drug use was associated with HCV exposure in male inmates as described in other studies with similar populations [22,35,36]. In a meta-analysis study, male inmates and injecting drug users were 24 times more likely to be HCV positive than non-injecting drug users [29]. Despite correctional system prohibitions against injection drug use, this practice occurs among male prisoners (2%). Even if the injection drug use may have been omitted by most inmates, because of the stigma, fears of recrimination and punishment, this risk factor was strongly associated with HCV exposure.

High-risk activities such as sharing of sharps for making tattoos and injection drug use are common practices carried out within the prisons [6]. Despite the prohibition, a few prisoners have access to tools for injection drug use, increasing the risk of acquiring blood-borne diseases such as HCV by sharing needles and syringes [37]. In this context, it is not surprising to find that, in our study, syringe and needle sharing remained independently associated with HCV exposure among male inmates.

The longer the incarceration is, the higher the risk of acquiring new infections due to inconsistent condom use or sharing of contaminated needles and syringes [38]. Long imprisonment periods and previous incarcerations may create even more opportunities for HCV acquisition and transmission [37,39]. Moreover, poor diagnosis and lack of knowledge regarding HCV transmission or medical treatment might influence the risk for HCV. In fact, this is consistent with our results which found association between HCV exposure and longer than 48-mounth incarceration period and, also observed new cases of HCV infection during the cohort study.

Moreover, this study demonstrated a high prevalence rate of HCV/HIV coinfection (11.1%) among male inmates probably due to the similarity in the transmission routes of these infections. Prisoners are known to engage in various activities that are considered risk factors for HCV/HIV coinfection, including high-risk sexual behaviors, sharing of needles/syringes and several types of paraphernalia for tattoos and drug use [16,40,41].

This is the first study of incidence of HCV infection involving Brazilian prison inmates. Despite of the low incidence rate, this data demonstrates that high-risk behavior already occur and highlight the importance of the prevention of this infection inside prisons [42,43]. The potential to identify the exact mechanism through which seven inmates were infected was limited by our study design, and by self-reported risk behaviors, such as drug use. [42,44]. Nevertheless, risk behaviors reported by new HCV cases should be interpreted with caution.

A study conducted in Scottish prisons found that the availability of needles and syringes at harm reduction programs for intravenous drug use, reflected in a lower incidence and thus lower prevalence of HCV [43]. Unfortunately, Brazilian prisons have not yet recognized the importance of access to effective prevention strategies to reduce the transmission of blood-borne infections in the facilities. This is because drug use prevention initiatives in prisons are implemented from a repressive perspective and not from a public health one [45].

Furthermore, the Brazilian criminal code includes neither death penalty nor life sentence. The prison system, with rare exceptions, fails to rehabilitate and reinsert prisoners into society. Indeed, a high rate of recidivism (above 60%) was found in our study starting the cycle anew. Therefore, due to the high turnover of inmates between prisons and the community, the epidemics of HCV infection combined with injection drug use among this population becomes a public health problem [46,47].

There were some strengths and limitations to this study. Some behavior risks may have been under-reported due to discrimination and stigma, leading to underestimating potential risk factors associated with HCV exposure. Moreover, this cross sectional survey was not designed to provide the temporal relationship between risky behaviors and HCV exposure in the male inmate population. Moreover, the small number of HCV exposure among female inmate population affected the analysis of risk factors among them. The limited number of positive cases in incidence study might also complicate the determination of risk factors. Despite these limitations, our study, having a large sample size, is representative of this population group and has important implications which would contribute to a clearer scenario of the most relevant risk factors for HCV exposure.

The new cases detected inside prisons reinforce the urgent need to plan effective prevention strategies and control HCV infection in the prison system environment in order to reduce the susceptibility for this infection, given the crowded conditions, uncontrolled exposure to illicit drugs and absence of serological screening at the time of imprisonment. The screening serological test and monitoring of IDUs in the prison system, making possible assess implementation of harm reduction strategies (exchange of syringes/needles). In addition, it is necessary to take health education measures directed to the prison population on risk factors associated to viral hepatitis. Finally, studies using phylogenetic analysis will also help to understand HCV transmission routes within this population.
